# Elevated Dengue Virus Nonstructural Protein 1 Serum Levels and Altered Toll-Like Receptor 4 Expression, Nitric Oxide, and Tumor Necrosis Factor Alpha Production in Dengue Hemorrhagic Fever Patients

**DOI:** 10.1155/2014/901276

**Published:** 2014-12-11

**Authors:** Denise Maciel Carvalho, Fernanda Gonçalves Garcia, Ana Paula Sarreta Terra, Ana Cristina Lopes Tosta, Luciana de Almeida Silva, Lúcio Roberto Castellano, David Nascimento Silva Teixeira

**Affiliations:** ^1^Institute of Health Sciences, Laboratory of Cellular Activation, Federal University of Triângulo Mineiro, 38025-350 Uberaba, MG, Brazil; ^2^Institute of Health Sciences, Department of Infectious and Parasitic Diseases, Federal University of Triângulo Mineiro, 38025-350 Uberaba, MG, Brazil; ^3^Human Immunology Research Group, Technical Health School, Federal University of Paraiba (UFPB), 58051-900 João Pessoa, PB, Brazil

## Abstract

*Background*. During dengue virus (DV) infection, monocytes produce tumor necrosis factor alpha (TNF-*α*) and nitric oxide (NO) which might be critical to immunopathogenesis. Since intensity of DV replication may determine clinical outcomes, it is important to know the effects of viral nonstructural protein 1 (NS1) on innate immune parameters of infected patients. The present study investigates the relationships between dengue virus nonstructural protein 1 (NS1) serum levels and innate immune response (TLR4 expression and TNF-*α*/NO production) of DV infected patients presenting different clinical outcomes. *Methodology/Principal Findings*. We evaluated NO, NS1 serum levels (ELISA), TNF-*α* production by peripheral blood mononuclear cells (PBMCs), and TLR4 expression on CD14^+^ cells from 37 dengue patients and 20 healthy controls. Early in infection, increased expression of TLR4 in monocytes of patients with dengue fever (DF) was detected compared to patients with dengue hemorrhagic fever (DHF). Moreover, PBMCs of DHF patients showed higher NS1 and lower NO serum levels during the acute febrile phase and a reduced response to TLR4 stimulation by LPS (with a reduced TNF-*α* production) when compared to DF patients. *Conclusions/Significance*. During DV infection in humans, some innate immune parameters change, depending on the NS1 serum levels, and phase and severity of the disease which may contribute to development of different clinical outcomes.

## 1. Introduction

Dengue virus (DV) infects 50–100 million people worldwide every year and an additional 2.5 billion people are at high risk, living in dengue endemic areas [1–3]. In Brazil, dengue fever (DF) has been a serious health problem and, in 2013, from the 2,351,703 cases reported in America, approximately 61% occurs in Brazil [2]. Dengue has various clinical presentations and clinical illness range from a self-limited dengue fever (DF) to the life-threatening syndromes of dengue hemorrhagic fever (DHF) and dengue shock syndrome (DSS), showing manifestations such as increased vascular permeability, hepatomegaly, decreased platelet counts, hemorrhage, and plasma leakage with the risk of fatal hypovolemic shock [4, 5].

Regardless of numerous studies, the immunopathological mechanisms involved in the development of severe dengue are not fully understood and various controversial results are being published around the world [5]. Antibody-dependent enhancement [6], inappropriate T cell [7, 8], “tsunami” cytokine response [9, 10], and host genetic factors [11] are amongst the postulated causes leading to severe dengue.

Monocytes and dendritic and endothelial cells seem to be the main targets of DV* in vivo* and* in vitro*, and DV antigens can be identified in macrophages of infected patients and also on endothelial cells of dead DHF patients [12–14]. Thus, it is apparent that interactions between monocytes and endothelial cells leading to a vascular damage play a key role in the pathophysiology of dengue disease. Monocytes/macrophages can produce various mediators in response to DV infection and it is possible that dysregulation of innate and bystander immune activation may play a role in aggravating disease. Among the mediators produced by activated monocytes, tumor necrosis factor alpha (TNF-*α*) and nitric oxide (NO) might be key molecules. A positive association between high soluble TNF receptor levels and the severity of DHF was described [15]. Single-nucleotide polymorphism analysis identified TNF-*α* polymorphisms at the TNF-308A allele to be a possible risk factor for development of hemorrhagic disease in patients infected with DV [16, 17]. Using a mouse model, a direct relationship between TNF-*α* and dengue hemorrhage was identified, because TNF-*α* deficiency greatly diminished hemorrhage development [18]. Moreover, production of NO can affect systemic vascular resistance and lead to hypotension, shock, and death if not corrected. NO levels are increased in many infectious diseases. When DVs were cocultured with human Kupffer or spleen cells, increased production of NO was reported [19], and elevated levels of NO were found in DF patients [20]. DVs were susceptible to a NO donor treatment and viruses were detected at higher rates in infected cells after iNOS inhibition, indicating that NO might play an important role in controlling monocytes DV infection [21]. Thus, it seems that TNF-*α* and NO would be involved not only in generating severe symptoms [22, 23] but also in the elimination of viruses [24–26].

TNF-*α* and NO are produced in response to toll-like receptor 4 (TLR4) stimulation. Toll-like receptors (TLRs) are important in microbial recognition [27] and they are involved in the generation of antiviral molecules and proinflammatory cytokines which probably exert immunopathological functions [27]. Although the implications of TLRs functions in viral infections have been investigated [28], the knowledge about dengue is restricted. de Kruif et al. [29] evaluated TLR gene-expression profiling of children with severe dengue infections. The authors demonstrated mainly that TLR7 gene transcription was upregulated, while TLR2 were downregulated, indicating the* in vivo* role of particular TLRs with different disease-severity parameters.

TLR4 is recognized as a LPS receptor [30, 31] and a previous study showed an interaction among DV, LPS, and CD14 at the membrane of primary human monocytes/macrophages [32]. The bacterial lipopolysaccharide (LPS), a ligand of the CD14-TLR4 complex, was able to block DV and modulate virally induced cytokine production by human monocytes and macrophages. So, based on that, we asked if there is a regulatory role for the LPS receptor, TLR4, on cytokine production during the acute phase of human DV infection.

DV genome is a single-stranded positive sense RNA which codes for 10 gene products, including structural proteins capsid (C), premembrane (prM), envelope (E), and nonstructural proteins NS1, NS2A, NS2B, NS3, NS4A, NS4B, and NS5 [33, 34]. Due to the fact that intensity of DV replication might influence clinical outcomes, it is important to investigate the impact of some viral proteins on innate immune parameters of DV infected patients. Among these proteins, the NS1 glycoprotein is a singular glycoprotein since it does not form part of the virion structure but is expressed on the membrane of infected cells. NS1 circulates at high levels in the sera of patients during the acute phase of illness, is a well-known early diagnostic marker, and may be involved on the pathophysiology of DV infection [35]. Preliminary evidence has shown that NS1 is involved in viral RNA replication [35], but an association between NS1 levels, perturbation of innate immune response, and severe disease is still unknown. In addition, toll-like receptor regulation on monocytes can also theoretically be elicited by proteins such as viral NS1. Thus, the aim of this study was to investigate the relationships between* in vivo* secreted levels of NS1 and innate immune response parameters (TLR4 expression and TNF-*α*/NO production) in infected dengue patients with different clinical outcomes.

## 2. Methodology

### 2.1. Study Group and Case Classification

DV infected patients (*n* = 37), 17 female and 20 male subjects, were enrolled in this study. Twenty healthy individuals, 10 females and 10 males, were included as healthy controls (HCs). All HCs tested negative for DV NS1 antigen and DV IgM/IgG antibodies and had not been vaccinated against yellow fever virus. Dengue patients were enrolled from February 2010 to April 2013 in UFTM University Hospital and two healthcare centers located in the city of Uberaba, Brazil. Dengue cases were classified as DF or DHF according to the 1997 World Health Organization (WHO) guidelines [36]. We applied the old guidelines since the new WHO guidelines published in 2009 are more directly focused on clinical practice and are not broadly used in research [37].

### 2.2. Blood Samples Collection and Processing

After 2nd (acute phase) and 9th day (beginning of convalescence phase) from the commencement of symptoms, twenty milliliters of heparinized peripheral venous blood (PB) and five milliliters without anticoagulant (serum) were collected from DV infected patients and noninfected HCs. The heparinized blood collected was used for isolation of peripheral blood mononuclear cells (PBMCs) and serum was stored at −80°C until further use.

### 2.3. Serological Diagnosis of Dengue

Dengue-specific IgM and IgG were detected using a capture ELISA (PanBio), whereas Dengue NS1 was detected using the immunochromatographic kit (Panbio, Queensland, Australia) according to manufacturer's instructions.

### 2.4. NS1 Serum Levels

Serum samples were collected at two different phases (acute and convalescence) from all patients and were subjected to diagnostic assays according to manufacturer's instructions. The qualitative presence of NS1 was determined using the Platelia NS1 Ag enzyme immunoassay (Bio-Rad Laboratories, Marnes-la-Coquette, France) as indicated by the manufacturer. For quantitative measurements the same kit was used but with a modified protocol also designed by the same manufacturer. According to Bio-Rad technical support version 05/2008 (Platelia Dengue NS1 Ag: quantitative detection of dengue virus NS1 antigen in human serum or plasma by enzyme immunoassay), due to its high sensitivity, about ninety percent (90%) of positive sera tested in routine with Platelia Dengue NS1 Ag qualitative are showing optical densities (OD) exceeding the assay range linearity (OD > 3.0). The quantitative protocol uses a different conjugate dilution (1 : 5000) and a formula for calculation of NS1 units based on recombinant human NS1 (positive control) and NS1 cut-off control kit calibrators OD readings. This protocol applies to samples that have been confirmed positive with the traditional protocol with an OD > 3.0 (or close to the maximum OD readable with the plate reader). Calculations for samples tested with diluted conjugate 1 : 5,000, NS1 Ag Bio-Rad units per milliliter (BRU/mL), are calculated by multiplying the OD of the sample tested with 1 : 5,000 diluted conjugate by 150 as follows:

(1) $$\begin{array}{c} \text{Sample}\;\text{NS}1\;\left(\text{BRU}/\text{mL}\right)=\text{Sample}\;\text{OD}\times \frac{150}{\text{R}4\text{m}}, \end{array}$$

where R4m is the mean value of the ODs of the cut-off control duplicates (R4).

Results are expressed in Bio-Rad units per milliliter (BRU/mL).

### 2.5. Isolation and Stimulation of PBMCs

PBMCs were isolated from heparinized blood samples of HCs and DV infected patients by density gradient centrifugation using Ficoll-Hypaque (Histopaque 1077, Sigma Aldrich Chemical Co., St. Louis, MO). PBMCs were cultured at 1 × 10^6^ cells/mL in 24-well polystyrene tissue culture plates at 37°C in 5% CO_2_, using RPMI 1640 medium (BioWhittaker, Walkersville, MD) supplemented with 10% heat-inactivated fetal bovine serum, 100 U/mL penicillin/streptomycin (Sigma Aldrich Chemical Co.), and 1% of L-glutamine (Sigma Aldrich Chemical Co.). The PBMCs were stimulated for 24 h in the presence or absence of a TLR4 agonist 10 *μ*g/mL of ultrapure lipopolysaccharide (LPS; InVivoGen, USA). PBMC culture supernatants were harvested after 18 h of cell culture and stored at −80°C until analysis. Aliquots of PBMCs were suspended in 1 mL of solution destined for freezing (90% inactivated fetal calf serum (FCS; Gibco, Invitrogen) plus 10% dimethyl sulphoxide (DMSO; Sigma Chemical Co., St. Louis, MO)) and stored initially at −80°C for 24 hr before introduction into liquid nitrogen, and aliquots were cryopreserved for later flow cytometer studies.

### 2.6. Cell Viability Determination

The viability of PBMCs in culture and after LPS stimulation was quantified by their ability to reduce 3-(4,5-dimethylthiazol-2-yl)-2,5-diphenyltetrazolium bromide (MTT) to formazan precipitate in duplicate wells. MTT (Sigma-Aldrich) at a final concentration of 5 mg/mL was added to each well 4 h before the termination of the experiment. Formazan dye was dissolved by incubation in DMSO (Merck, Berlin, Germany) and its concentration was determined spectrophotometrically at an absorbance wavelength of 560 nm.

### 2.7. TNF-*α* Quantification

PBMC culture supernatants were harvested after 18 h of culture and tumor necrosis factor alpha (TNF-*α*) concentration was measured by ELISA according to the manufacturer's protocol (BD-Bioscience, USA).

### 2.8. Serum Nitric Oxide Measurement

The concentration of NO in serum samples was indirectly measured by determining both nitrate and nitrite levels. Total serum NO was measured by utilizing a nitric oxide (NO_2_^−^/NO_3_^−^) assay kit (Sigma Aldrich, USA), following the manufacturer's instructions. This assay determines nitric oxide based on the enzymatic conversion of nitrate to nitrite by nitrate reductase. The reaction is followed by a colorimetric detection of nitrite as a product of the Griess reaction, based on the diazotization reaction in which acidified NO_2_^−^ produces a nitrosating agent, which reacts with sulfanilic acid to yield the diazonium ion. This ion is then combined to* N*-(1-naphthyl) ethylenediamine to form the chromophoric azo derivative which absorbs light at 540 nm. Protein interference was avoided by treating samples with zinc sulfate and centrifugation for 10 min at 2000 ×g. Samples were spectrophotometrically quantified using a Turner microplate reader at 540 nm (PROMEGA, USA), NaNO_2_ was used as a standard, and a curve of nitrite concentration against its OD was plotted.

### 2.9. Flow Cytometry

PBMCs flow cytometry was performed by incubating 50 *μ*L containing 5 × 10^5^ PBMCs with optimal concentrations of monoclonal antibodies: anti-CD14-FITC and anti-TLR4-PE for 30 min, at room temperature in the dark. Cells were washed twice (300 ×g for 5 minutes) and suspended in 500 *μ*L of PBS with 1% of fetal calf serum (FCS) and 0.1% sodium azide for data acquisition. Before each experiment, the instrument was checked for stability and reproducibility using Calibrite beads (Becton Dickson, San Jose, CA). Control tubes include cells incubated with medium alone (control of background fluorescence) and cells incubated with FITC and PE conjugated mouse isotype control antibodies (control of nonspecific binding). During acquisition, a hundred thousand events were counted per sample.

### 2.10. Cytometry Analysis Strategy

The expression of the TLR4 was defined by using gates on contour plots. CD14^+^ cells were obtained by drawing a gate of fluorescence versus specific granularity (SSC parameter). The cells contained in this gate, named region 1 (R1), were further analyzed in a PE-fluorescence channel representing the TLR4 expression.

### 2.11. Statistical Analyses

All data analyses were carried out using the applicative GraphPad Prism software (Graph Pad, CA). As the numeric variables had nonparametric distribution, Mann-Whitney and Kruskal-Wallis tests were used to compare two or more groups, respectively. The significance set was adjusted to *P* < 0.05.

## 3. Results

### 3.1. Study Group Characterization

In this study, 57 patients were enrolled, 37 of which were positive for DV infection. From the 37 dengue positive cases, 11 were classified as DHF patients. The demographic and clinical information of the 37 dengue patients enrolled in this study are summarized in Table 1.

### 3.2. NS1 Quantitation in Patient's Serum

**Figure 1:**
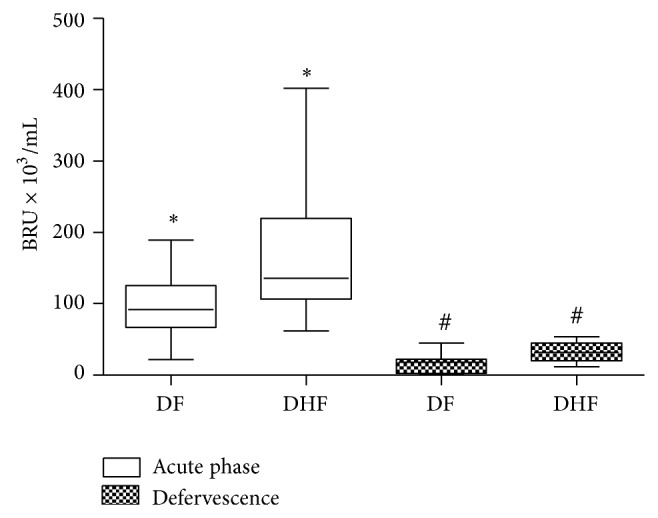
Dengue virus nonstructural protein 1 (NS1) serum levels of patients with dengue fever (DF, *n* = 26) and dengue hemorrhagic fever (DHF, *n* = 11) during the acute phase and convalescence, measured by ELISA and expressed as median BRU × 10^3^/mL in a box plot. Equivalent symbols (∗, #) represent statistically significant differences (*P* < 0.05) between groups.

NS1 antigen was found circulating from the second day after the onset of fever to day 9. NS1 circulation levels varied among individuals during the course of the disease, ranging from 2.2 to 600 BRU/mL of serum. During the acute febrile phase, DHF patients display higher NS1 serum levels than DF patients. Moreover, during the beginning of convalescence phase, NS1 levels were also higher on DHF than in DF patients (Figure 1).

### 3.3. Nitric Oxide Serum Levels and Clinical Outcomes

**Figure 2:**
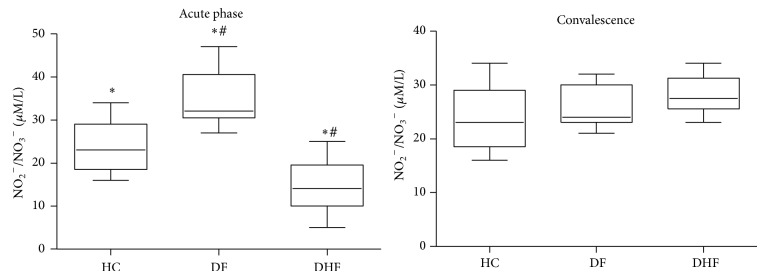
Nitric oxide serum levels (*μ*M of NO_2_^−^/NO_3_^−^) of patients with dengue fever (DF, *n* = 26), dengue hemorrhagic fever (DHF, *n* = 11), and healthy controls (HC, *n* = 20) measured by Griess reaction after reduction of serum samples with nitrate reductase. Results were expressed as median using a box plot. Equivalent symbols (∗, #) represent statistically significant differences (*P* < 0.05) between two groups.

During the acute febrile phase of DV infection, we observed a significant increase in NO serum levels on DF patients' serum and a decrease in DHF patients when compared to healthy controls (Figure 2). During the convalescence phase no differences could be detected among groups.

### 3.4. Nitric Oxide and NS1 Serum Levels

**Figure 3:**
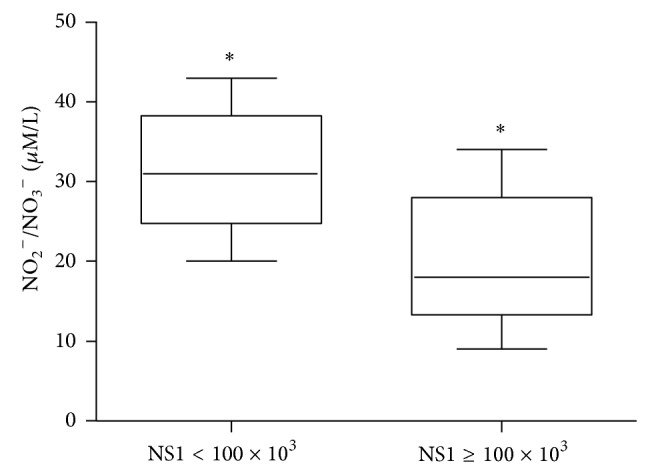
Nitric oxide serum levels (*μ*M of NO_2_^−^/NO_3_^−^) of dengue infected patients (*n* = 37) during the acute phase of the disease presenting low (NS1 < 100 × 10^3^ BRU/mL) and high (NS1 ≥ 100 × 10^3^ BRU/mL) dengue virus nonstructural protein 1 (NS1) serum levels measured by Griess reaction after reduction of serum samples with nitrate reductase. Results were expressed as median using a box plot. Equivalent symbols (∗) represent statistically significant differences (*P* < 0.05) between groups.

When analyzing the distribution of NS1 serum levels among all DV infected patients we observed a clear division between two patient groups (data not show). One group displayed low levels of serum NS1 (below 100 × 10^3^ BR Units/mL) and the other displayed high levels of NS1 (equal or above 100 × 10^3^ BR Units/mL). Thus, besides the clinical form and based on the fact that we establish a cut-off, we also analyzed patient's data according to their NS1 content (<100 × 10^3^ BR Units/mL or ≥100 × 10^3^ BR Units/mL). We show that patients with NS1 serum levels ≥100 BR × 10^3^ BR Units/mL displayed reduced NO serum levels when compared to patients with NS1 levels below 100 × 10^3^ BR Units/mL (Figure 3).

### 3.5. Stimulation of TLR4, TNF-*α* Production, and NS1 Levels and Clinical Outcomes

**Figure 4:**
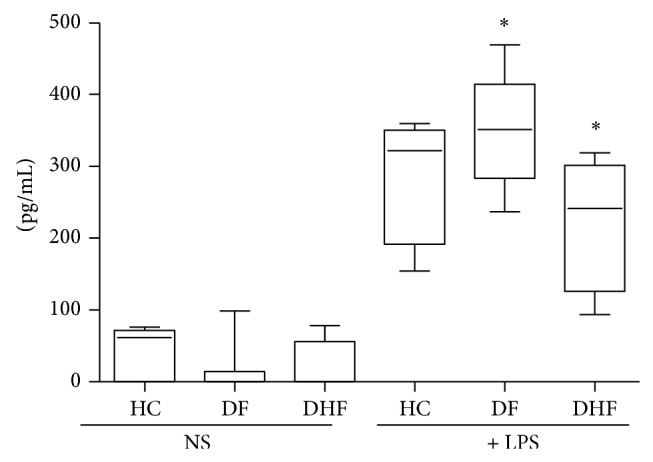
Tumor necrosis factor alpha (TNF-*α*) production (pg/mL) by 1 × 10^6^ peripheral blood mononuclear cells (PBMCs) from patients with dengue fever (DF, *n* = 26) and dengue hemorrhagic fever (DHF, *n* = 11) during the acute phase of the disease and healthy controls (HC, *n* = 20). PBMCs were not stimulated (NS) or stimulated for 18 hours with 10 *μ*g/mL of lipopolysaccharide (+ LPS) and TNF-*α* production measured by ELISA. Results were expressed as median using a box plot. Equivalent symbols (∗) represent statistically significant differences (*P* < 0.05) between groups.

**Figure 5:**
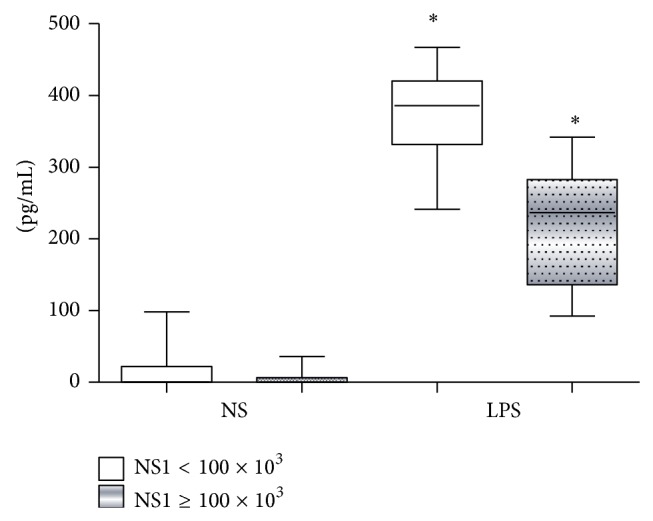
Tumor necrosis factor alpha (TNF-*α*) production (pg/mL) by 1 × 10^6^ peripheral blood mononuclear cells (PBMCs) from dengue infected patients (*n* = 37) during the acute phase of the disease presenting low (NS1 < 100 × 10^3^ BRU/mL) and high (NS1 ≥ 100 × 10^3^ BRU/mL) NS1 serum levels. PBMCs were not stimulated (NS) or stimulated for 18 hours with 10 *μ*g/mL of lipopolysaccharide (LPS) and TNF-*α* production measured by ELISA. Results were expressed as median using a box plot. Equivalent symbols (∗) represent statistically significant differences (*P* < 0.05) between groups.

Production of TNF-*α* after TLR4 stimulation with LPS was also investigated. DHF patients present a lower response to TLR4 stimulation than DF patients (Figure 4).  Figure 5 shows that dengue patients with higher NS1 serum levels displayed a reduced TLR4 response to LPS (with a lower TNF-*α* production) than the group of patients with less NS1 content (<100 × 10^3^ BR Units/mL).

### 3.6. TRL4 Expression on CD14^+^ Cells

**Figure 6:**
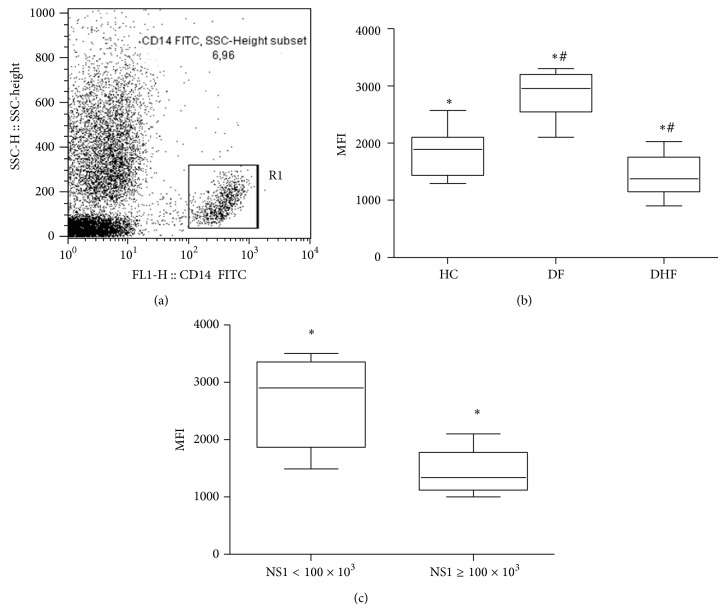
Toll-like receptor (TLR-4) expression of CD14^+^ cells from patients (*n* = 37) infected with dengue virus during the acute phase of the disease. (a) represents the gate strategy: a gate was drawn on SSC × FL1/CD14^+^ cells. (b) represents anti-TLR4-PE mean fluorescence intensity (MFI) of CD14^+^ cells from dengue fever (DF, *n* = 26), dengue hemorrhagic fever (DHF, *n* = 11), and healthy controls (HC, *n* = 20). (c) represents anti-TLR4-PE MFI of CD14^+^ cells from dengue infected patients with low (NS1 < 100 × 10^3^ BRU/mL) and high (NS1 ≥ 100 × 10^3^ BRU/mL) dengue virus nonstructural protein 1 (NS1) serum levels. Results on (b) and (c) graphs were expressed as median using a box plot. Equivalent symbols (∗, #) represent statistically significant differences (*P* < 0.05) between groups.

Subsequently to the alterations detected on TLR4 response to its agonist LPS in DHF patient's cells, we attempt to investigate if this reduced response was due to modulations on TLR4 expression on monocytes. In fact, TLR4 expression was downmodulated on DHF monocytes during the acute phase of the disease when compared to CD14^+^ cells obtained from DF patients (Figure 6(b)). High levels of serum NS1 were also associated with a reduced TLR4 membrane expression on these cells (Figure 6(c)).

## 4. Discussion

It is possible that during DV infection, protection or pathogenesis is determined at the edge of innate and adaptive immunity controlled by cytokines produced as a consequence of DV interactions with its main targets, human monocytes and endothelial cells [12–14]. Besides that, it is important to know the impact of viral proteins, such as NS1, on innate immune parameters of DV infected patients. However, a defined connection between viral replication and increased vascular permeability in DHF development is still subject of speculation.

We are able to detect higher serum levels of soluble NS1 in DHF patients' serum when compared to DF (Figure 1). NS1 concentrations must be a key point, since high levels of this protein could affect innate immune response and may influence later development of different clinical forms. One study demonstrated that NS1 levels in human sera appear to be significantly higher in patients who developed DHF rather than DF [38]. During DV infection, mouse and humans develop antibodies against NS1 that cross-react with endothelial cell epitopes inducing a nitric oxide dependent apoptosis [39]. Results from our work and data from other studies strengthen the fact that intensity of DV replication in the early times of infection may influence clinical outcomes, but pathogenesis of endothelial dysfunction related to vascular leakage syndrome is not a fully understood phenomenon.

Among important innate immune parameters that could be affected by dengue NS1 levels, NO and TNF-*α* seem to be very important molecules. Nitric oxide, a gaseous molecule, is a product of enzymes called NO synthases (NOS) [40] that are classified into three isoforms, specifically, endothelial NOS (eNOS), inducible NOS (iNOS), and neuronal NOS (nNOS). The main physiologic function of eNOS-derived NO is vasodilatation. iNOS can be found in some cell types, such as monocytes/macrophages as well as endothelial cells, and is expressed when cells are activated with molecules such as LPS or interferon gamma (IFN-*γ*) [41]. NO produced by macrophages and endothelial cells plays an important role in regulating the diameter of blood vessels, inhibiting leukocyte adhesion and platelet aggregation [40, 41].

NO is probably involved in hemorrhagic fevers and virus-induced shock when produced in high amounts [42]. In our study, we found decreased NO serum levels during febrile acute phase in DHF compared to DF patients (Figure 2). The reasons for the lower levels of NO in DHF patients are not clear to us but damage of endothelial cells during the acute phase of DV infection, with a consequent failure of endothelial NO synthase, could be involved. On the other hand, since NO is a “double-sword” molecule [43], the increased levels of NO in DF could play a role in decreasing viral load and altering the evolution of the disease to its hemorrhagic form. Interestingly, patients with low NS1 serum levels during the acute phase of the disease also displayed higher NO serum levels (Figure 3). The pattern of low serum NO levels in the DHF group during the acute febrile phase and higher serum NO levels during the beginning of convalescence phase may also be due to different eNOS and iNOS modulations in each phase, but this hypothesis needs to be tested. A previous study by Levy et al. showed that distinct dengue serotypes yield similar NO levels [44], suggesting that NO appears to be involved in the progression of dengue infection independent of the DV serotype. Taken together, these data demonstrate that NO might be contributing not only to protection but also to pathology of dengue infection, depending on the amount of NO produced and the phase of the disease.

Activated monocytes/macrophage also produce TNF-*α* after TLR4 stimulation. Thus, modulation of TLR4 expression and responsiveness is an important issue to investigate during human DV infection. A correlation between high concentration TNF-*α* in blood and the severity of DHF has been reported [15–17]. TLR functions as receptors during dengue infection are not yet clear and our data indicates a differential regulation of TLR4 expression (Figure 6(b)) and responsiveness (Figure 4) during the acute phase of this illness on cells from DHF when compared to DF patients and that may be also affected by NS1 serum levels (Figure 6(c)). Data from the literature shows that DV cell entry in the monocyte cell line THP-1 is facilitated by antibodies, activation of TLR-negative regulators, and downregulation of TLR4 and other genes associated with TLR signaling [45]. Besides that, entry of DV via Fc receptor, during a virus-enhancing antibody complex infection, preferentially switches off the TLR4 dependent signaling, due to a significant collapse of the TLR4 pathway [46]. The following downregulation of the proinflammatory cytokine production may provide a growth advantage for DV to propagate in host macrophages and for the development of more serious forms of the disease.

The relationships between* in vivo* dengue virus nonstructural protein 1 (NS1) levels and innate immune response parameters (TLR4 expression and TNF-*α*/NO production) in infected dengue patients were investigated in our work. Results obtained indicate that DHF patients displayed an altered innate immune response, with low TLR4 expression, and reduced NO and TNF-*α* production during the acute phase of the disease when compared to DF patients. Thus, our data suggests that, during this early acute phase, patients who develop DHF may present a less efficient antiviral innate immune response to DV that may promote an accumulation of huge amounts of circulating NS1 protein (Figure 1), which in turn may affect these correlated innate immune parameters (NO, TNF-*α*, and TLR4; Figures 3, 5, and 6, resp.).

A differential expression and responsiveness of TLR4 on CD14^+^ cells in DHF patients could be one relevant factor that leads to different clinical outcomes, but new studies are necessary to understand the precise role of these pathways on DHF development in humans.

## 5. Conclusions

During DV infection in humans, DHF patients display alterations on innate immune response (expression and responsiveness of TLR4 on CD14^+^ cells and TNF-*α*/NO production) that are inversely correlated to NS1 serum levels and phase and severity of the disease, which may contribute to development of different clinical outcomes.

## Figures and Tables

**Table 1:** Demographic and clinical information of dengue patients and healthy controls enrolled in the study.

Patients data	Healthy controls (HC) (*n* = 20)	Dengue fever (DF) (*n* = 26)	Dengue hemorrhagic fever (DHF) (*n* = 11)
Age (mean ± SD)	37 ± 14	31 ± 11	39 ± 22
Male (*n* = 30)	10	14	06
Females (*n* = 27)	10	12	05
Platelets (×10^3^/mm^3^)	271 ± 36	152 ± 82	68 ± 27
